# Retraction: Lee et al. Nurses’ Attitudes Toward Psychiatric Help for Depression: The Serial Mediation Effect of Self-Stigma and Depression on Public Stigma and Attitudes Toward Psychiatric Help. *Int. J. Environ. Res. Public Health* 2020, *17*, 5073

**DOI:** 10.3390/ijerph19073856

**Published:** 2022-03-24

**Authors:** Eunmi Lee, Yoo Mi Jeong, Su Jeong Yi

**Affiliations:** 1 Department of Nursing, Research Institute for Basic Science, Hoseo University, Asan-si 31499, Korea; sweetbear2@hanmail.net; 2 College of Nursing, Dankook University, Cheonan-si 31116, Korea; 12181056@dankook.ac.kr

The published article [1] has been retracted at the request of the authors. Following its publication, the authors contacted the editorial office regarding data used without authorization. This issue was confirmed by the author’s Institutional Review Board (IRB).

Adhering to our complaints procedure, an investigation was conducted and the article is therefore retracted.

This retraction was approved by the Editor in Chief of the journal.

The authors agreed to this retraction.
